# Practice facilitation to implement stepped care for unhealthy alcohol use in HIV clinics: study protocol for a type III hybrid effectiveness-implementation study

**DOI:** 10.1186/s13722-026-00662-6

**Published:** 2026-03-31

**Authors:** Geetanjali Chander, D. Scott Batey, Elizabeth J. Austin, Madeleine J. Bentley, Edward R. Cachay, Heidi M. Crane, Claire Farel, Julia Fleming, David J. Grelotti, Heidi E. Hutton, Bulat Idrisov, JoAnna Mathena, Mary E. McCaul, Sonia Napravnik, Conall O’Clerigh, Bryan Lau, Emily C. Williams

**Affiliations:** 1 Department of Medicine, School of Medicine, University of Washington, 325 9th Avenue, Seattle, WA USA; 2 School of Social Work, Tulane University, New Orleans, LA USA; 3 Department of Health Systems and Population Health, School of Public Health, University of Washington, Seattle, WA USA; 4 Department of Medicine, University of California San Diego School of Medicine, San Diego, CA USA; 5 Department of Medicine, University of North Carolina Chapel Hill, Chapel Hill, NC USA; 6 Fenway Community Health Center, Boston, MA USA; 7 Department of Psychiatry, San Diego School of Medicine, University of California, San Diego, CA USA; 8 Department of Psychiatry and Behavioral Sciences, Johns Hopkins University School of Medicine, Baltimore, MD USA; 9 Department of Psychiatry, Massachusetts General Hospital, Harvard Medical School, Boston, MA USA; 10 Department of Epidemiology, Johns Hopkins Bloomberg School of Public Health, Baltimore, MD USA; 11 Center of Innovation for Veteran-Centered and Value-Driven Care, Health Services Research & Development, VA Puget Sound, Seattle, WA USA

**Keywords:** Alcohol, Screening, Alcohol use disorder treatment, Practice facilitation, HIV

## Abstract

**Background:**

Despite availability of evidence-based alcohol reduction interventions (EBIs), unhealthy alcohol use (UAU) remains a barrier to HIV medication adherence, viral suppression, and retention in HIV care. While translation of alcohol EBIs into HIV clinical practice is important for comprehensive HIV care, their implementation in HIV settings is impeded by resource constraints, workflow challenges, and negative perceptions of alcohol-related care.

**Methods:**

Guided by the Consolidated Framework for Implementation Research and Reach, Effectiveness, Adoption, Implementation and Maintenance frameworks, we will conduct a Hybrid Type 3 effectiveness-implementation study testing whether external practice facilitation increases reach, adoption, implementation, and maintenance of stepped care for UAU in three Center for AIDS Research (CFAR) Network of Integrated Clinical Systems (CNICS) HIV clinics in the United States. We will secondarily test whether practice facilitation is associated with clinical outcomes. We will first conduct a mixed methods formative evaluation to tailor delivery of practice facilitation (including the tools, technical assistance, and content expertise offered) based on each site’s context and needs (Aim 1). We will then deliver practice facilitation across the three sites sequentially to implement a stepped-care model of alcohol treatment to patients with UAU. Stepped care, where non-responders to alcohol EBIs are offered a more intensive therapy, will specifically include person- or computer-delivered brief alcohol intervention, on-line cognitive behavioral therapy, and linkages to alcohol pharmacotherapy. Clinical outcomes will include: (1) clinic-level implementation outcomes of stepped care EBIs for alcohol use including reach, adoption, maintenance, using mixed methods (Aim 2a) and (2) patient-level outcomes using interrupted time series analysis with synthetic controls (Aim 2b). Finally, we will use summative evaluation to describe barriers and facilitators to implementation of the interventions at each site to describe maintenance and inform widespread sustainable implementation (Aim 3).

**Discussion:**

This trial tests an implementation strategy to improve the delivery of stepped care, an evidence-based treatment approach for UAU in HIV clinics. Practice facilitation has shown promise for implementing evidence-based care for UAU in primary care but use of practice facilitation for this purpose in HIV clinics is novel. Results from this implementation study may support broader implementation of alcohol evidence-based practices in HIV care.

**Trial registration:**

The trial is registered with Clinicaltrials.gov, identifier NCT05241990 Date of submission 2/16/2022.

## Background

At-risk or unhealthy alcohol use (UAU), a spectrum of alcohol use ranging from exceeding weekly or daily recommended limits to alcohol use disorder (AUD) [1], is prevalent among persons with HIV (PWH), with over 25% endorsing patterns of unhealthy use [2]. UAU disrupts critical steps in the HIV Care Continuum, including antiretroviral therapy (ART) use, ART adherence, and viral suppression [3–7]. UAU also complicates the management of other medical conditions, including cardiovascular, and liver disease, and increases the risk for certain malignancies, including breast, colon, and head and neck cancer [8]. Given that UAU is a modifiable barrier to optimal clinical outcomes among PWH, increasing access to and uptake of evidence-based alcohol reduction strategies is an essential component of comprehensive HIV care.

Though alcohol screening and brief intervention are recommended for primary care [9], most patients do not receive these services [10, 11]. Further, most people in need of alcohol treatment do not access traditional treatment services citing barriers of cost, concerns of stigma or a negative effect on their job, and lack of knowledge of where to get treatment [12, 13]. As such, integration of evidence-based interventions (EBIs) for alcohol reduction in HIV clinics may address an important need and help to overcome common barriers. Studies among PWH support brief alcohol intervention (computer and person-delivered), motivational interviewing, cognitive behavioral therapy, and pharmacotherapy as effective EBIs for reducing alcohol use [14–21]. In a study conducted in two university-based HIV clinics (Birmingham, Alabama and Seattle, Washington), tablet-based alcohol screening combined with a computer-delivered brief intervention significantly reduced drinks per week among PWH with UAU [22]. In addition, stepped care, where non-responders to initial treatment were escalated to more intensive approaches, was effective in reducing alcohol use and improving viral suppression among United States (US) Veterans with HIV and AUD [23].

Implementation of EBIs for UAU in clinical settings is challenged by provider- and clinic-level barriers including lack of time and resources, inconsistent screening, inadequate training in managing UAU, operational workflows, and low confidence in the use of alcohol pharmacotherapy [24]. Practice facilitation is a multilevel intervention implementation strategy that addresses barriers to implementation of evidence-based practices. With practice facilitation, a practice coach offers tools, resources, hands-on guidance, and content expertise to assist the team in developing strategies to successfully implement an evidence-based practice [25–27]. Support by a practice coach is provided in near-real time and optimized by relationship development with each clinic site. There is substantial evidence in favor of practice facilitation in primary care, and multiple studies are using practice facilitation to implement alcohol EBIs [23, 25, 27–30]. Based on evidence in primary care settings [31, 32], practice facilitation for implementing alcohol EBIs may hold great promise for improving access to care in HIV clinics.

We are conducting a novel study in HIV clinics in which we will test practice facilitation to implement stepped care for UAU. In this manuscript, we describe the protocol of our Hybrid Type 3 effectiveness-implementation study guided by the RE-AIM (Reach, Effectiveness, Adoption, Implementation and Maintenance) evaluation framework [33]. Our study tests whether external practice facilitation increases reach, adoption, implementation, and maintenance of stepped care for UAU (our clinical intervention) in three Center for AIDS Research (CFAR) Network of Integrated Clinical Systems (CNICS) HIV clinics. We will secondarily test whether practice facilitation is associated with decreased UAU and improved ART adherence and viral suppression at the patient level (**e**ffectiveness). Formative and summative evaluations – guided by the Consolidated Framework for Implementation Research (CFIR) [34] - will also be used to tailor the implementation supports provided throughout the trial and characterize barriers and facilitators to implementation.

## Methods

This protocol follows reporting procedures outlined by the Standards for Reporting Implementation Studies (StaRI) statement [35].

### Implementation context

All implementation activities are nested within CNICS, a longitudinal, multi-site clinical cohort study of adults ≥ 18-years-old with HIV enrolled in care. An electronic health record (EHR)-based network, CNICS integrates clinical data from a large and diverse population of PWH across ten academically-affiliated HIV clinics in the US [36].CNICS prospectively collects comprehensive patient data, including validated health outcomes, across a range of diseases, as well as detailed patient reported outcomes (PROs) and behaviors at the time of clinical visits [37]. Through regular computer-based PRO administration, delivered at approximately six-month intervals, patients report on symptoms and behaviors, including alcohol use, using the Alcohol Use Disorders Identification Test-Consumption (AUDIT-C), the AUDIT, and the MINI International Neuropsychiatric interview alcohol domain (MINI) [38–40], with responses summarized and shared with providers. The PROs provide a foundation on which to embed EBIs into clinical care sites and to collect outcomes. CNICS sites have staff responsible for administration of the PRO who can link individuals to an intervention. Activities for the current study occur at three geographically diverse CNICS sites located in Boston, Massachusetts, Chapel Hill, North Carolina, and San Diego, California with a prevalence of unhealthy alcohol use ranging from 23% to 33%.

### Conceptual framework and logic model

**Fig. 1:**
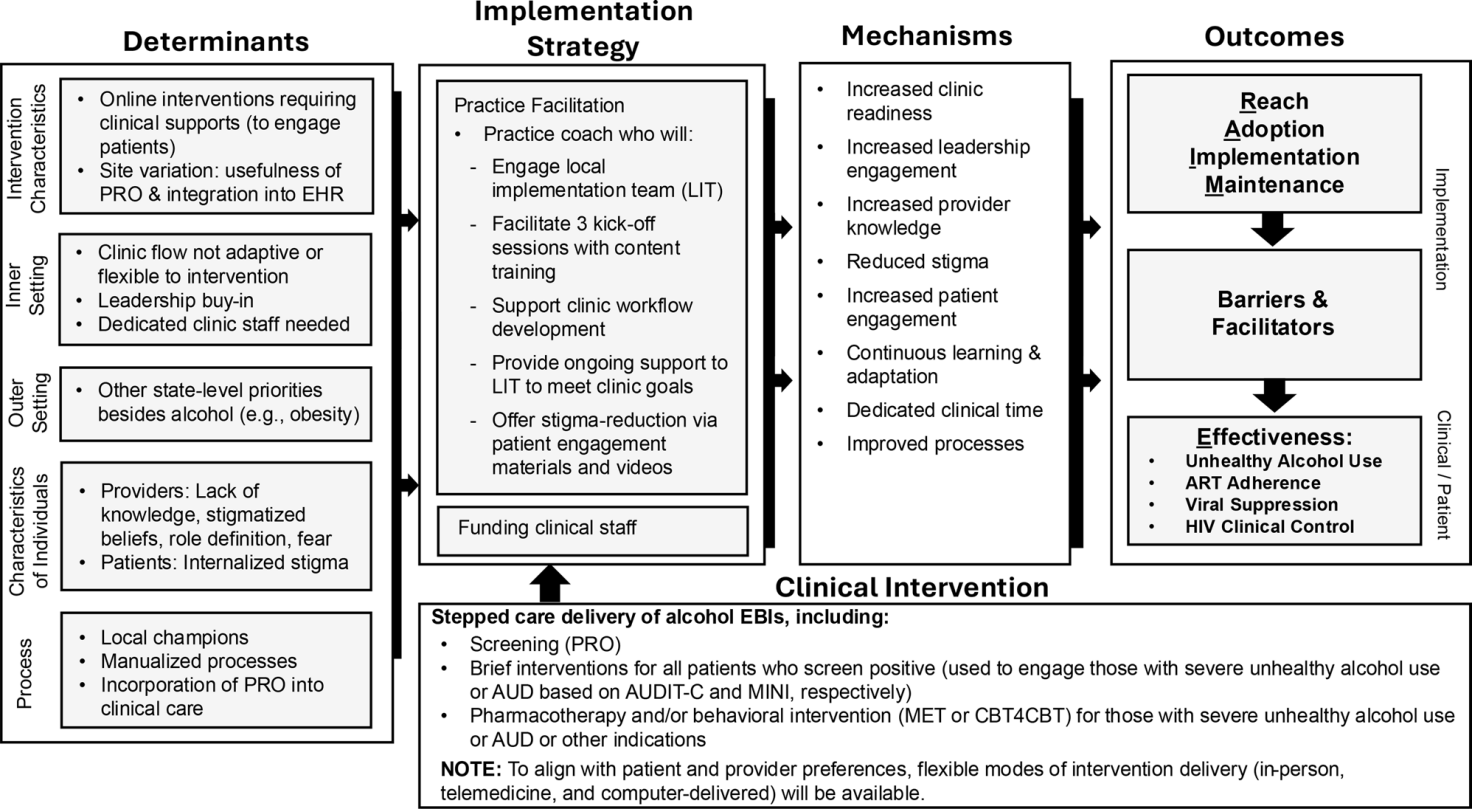
Revised logic model, based on implementation learnings

The design of this implementation study is guided by the Consolidated Framework for Implementation Research (CFIR) and the RE-AIM evaluation framework [41–43]. The CFIR identifies five domains of implementation and several sub-domains which can be used to identify implementation barriers, devise strategies to optimize implementation, and evaluate the effectiveness of implementation efforts [33]. The domains encapsulated in the CFIR and depicted as “determinants” in Fig. 1 include: (1) characteristics of the clinical intervention, (2) individuals using it, the (3) outer and (4) inner settings in which implementation occurred, and (5) the implementation process. In alignment with Smith et al.‘s methodology [44] for planning, executing, reporting, and synthesizing implementation projects, our framework applies the determinants to all aspects of the study, including the implementation strategy we have chosen (practice facilitation), barriers and facilitators to implementation overall, the success of the implementation strategy particularly, and the mechanisms through which we anticipate that practice facilitation will foster successful implementation of the clinical intervention, alcohol EBIs. The RE-AIM framework is applied to ascertainment of our outcomes, which are also determined by the CFIR domains through implementation work.

### Implementation strategy: practice facilitation

In this Hybrid Type 3 implementation-effectiveness study [45], we are testing practice facilitation as the implementation strategy. Practice facilitation uses multiple discrete implementation strategies supported by a practice coach (internal or external) who offers flexible supports throughout the implementation period that are tailored to local context (e.g., to each site) [46, 47]. The primary objective of practice facilitation is to foster internal capacity for change within a clinical setting. In practice facilitation, practice coaches assist clinical teams in identifying gaps in care and facilitate the team’s development of a plan to address those gaps. This often involves activities of education or knowledge brokering, technical assistance, coaching, expert consultation, and other supports as identified by the local site [48].

For this study, we are providing the three clinics with an external Practice Coach, a person outside of their organization who has expertise in alcohol-related care, practice improvement approaches, and implementation science. The same practice coach will work with each site. In addition, sites will assemble a Local Implementation Team (LIT), an interdisciplinary group of local clinical and administrative champions with the shared goal of implementing the clinical intervention. While each LIT will be expected to have a combination of clinical and administrative research champions, the specific membership of and communication pathways used with each LIT may vary based on the local context of each site.

Practice facilitation will be delivered to each clinic over an 18-month implementation period. The implementation period is organized around three phases of implementation: *pre-implementation*, *implementation*, and *sustainment*. The Practice Coach and LIT will collaborate on implementation activities via regular teleconference meetings and ad hoc activities as described in Table 1, however activities will vary based on each site’s needs, preferences, and infrastructures. To promote continuous learning and adaptation by the implementation team, each clinic’s implementation period is staggered across the study so that their *pre-implementation* phases begin approximately one year apart from one another.

**Table 1:** Implementation and practice facilitation activities over the implementation period

Implementation Phase	Implementation Objectives	Anticipated Practice Coach Activities
*Pre-implementation* (3 months)	• Adapt workflows and/or clinic resources to accommodate delivery of stepped care for UAU • Train & prepare staff on clinical delivery of stepped care for UAU	• Facilitation of bi-weekly meetings with LIT • Identification and preparation of local champions • Identification, development, and/or delivery of training resources • Facilitation of design session and/or focused meetings to design and/or refine workflows for delivery of stepped care for UAU
*Implementation* (12 months)	• Deliver stepped care for UAU within clinic	• Facilitation of monthly meetings with LIT • Real-time delivery of monitoring and feedback data on implementation progress • Facilitation of process improvement approaches (e.g., Plan, Do, Study, Act (PDSA)) • Technical assistance to design and/or refine implementation support materials (e.g., patient and/or provider-facing materials that support implementation)
*Sustainment* (3 months)	• Establish plan for long-term integration and monitoring of stepped care practices in clinic	• Facilitation of monthly meetings with LIT • Technical assistance to develop sustainment plans that address the person, technical, and leadership resources needed to sustain stepped care long-term

### Clinical intervention

The clinical intervention (stepped care) includes clinic-based alcohol use screening, followed by alcohol reduction EBIs delivered through a stepped care approach.

#### Screening

As part of routine clinical care, PWH undergo tablet-based, PRO screening with the AUDIT-C approximately every six months. Based on validated cut-points (AUDIT-C scores ≥ 3 for women or ≥ 4 for men), administration of the 10-item AUDIT will be triggered in the PROs. Women with AUDIT scores ≥ 5 and men ≥ 6 then receive the MINI International Neuropsychiatric Interview alcohol domain (MINI) [38] to assess for AUD. When a patient screens positive for UAU, a staff member designated by the site will receive an automated alert via pager with the recommended intervention based on UAU severity. The patient is then approached, consented, and linked to an EBI if they are interested. This system has been programmed into the CNICS PRO platform from earlier work [22].

#### Interventions

**Fig. 2:**
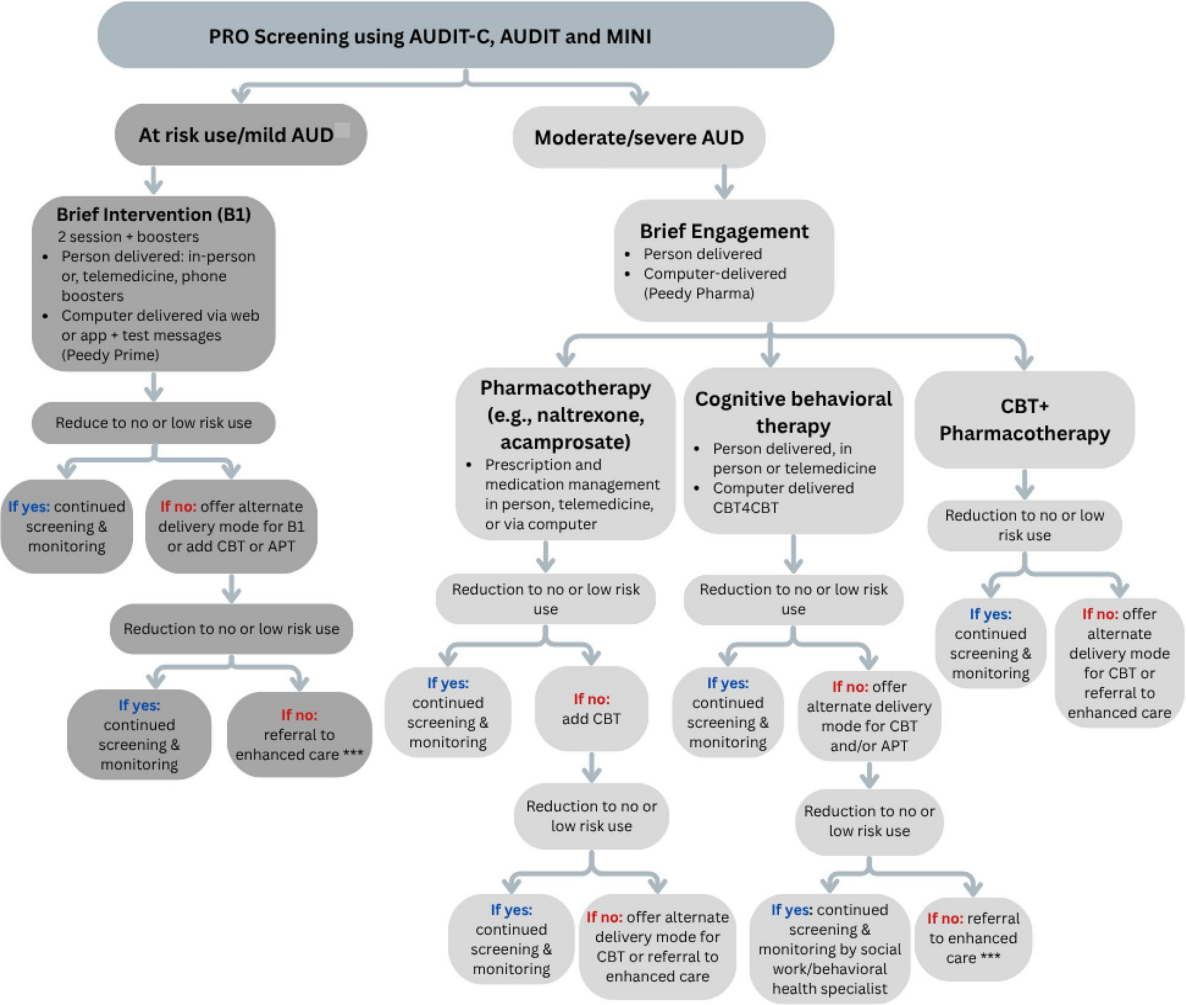
Alcohol stepped care approach

(see Fig. 2). The specific alcohol EBI offered to each patient is determined by the absence or presence of AUD and AUD severity. Individuals with UAU and either no AUD or mild AUD are offered a brief alcohol intervention, while individuals with moderate to severe AUD are offered alcohol pharmacotherapy and cognitive behavioral therapy in addition to a brief intervention. The alcohol EBIs include computer (CBI) [22]- or person-delivered brief intervention, on-line cognitive behavioral therapy (CBT4CBT) [49], and alcohol pharmacotherapy. The CBIs, described in our prior work [21], use cognitive behavioral techniques for alcohol reduction or cessation. The sessions are delivered in a motivational interviewing style by a 3-D virtual counselor. Four sessions tailored to levels of alcohol severity and session number (initial vs. follow-up) (Table 2) have been developed. All CBI sessions have also been scripted to be delivered in-person and by Zoom. CBT4CBT is an online cognitive behavioral therapy program consisting of seven modules, 45 min each in length, which include refusal skills, recognizing and coping with cravings, addressing thoughts about alcohol use, patterns of thinking, and decision making around alcohol use. Finally, alcohol pharmacotherapy includes Federal Drug Administration (FDA) approved therapies for alcohol use, including naltrexone and acamprosate.

**Table 2:** Description of computer-delivered interventions and pharmacotherapy

Intervention	Inclusion	Description
CBI Prime	PWH with UAU or mild AUD at visit 1	Includes education about standard drink sizes, patterns/spectrum of alcohol use, decisional balance exercises, and the impact of alcohol on HIV, comorbidities, and medication adherence. It also presents common moods or situations for drinking and offers strategies for coping with these. Patients have the opportunity to set drinking goals
CBI Pharma	PWH with moderate/severe AUD at visit 1	Includes CBI Prime content, an overview of pharmacotherapy options, and how they may help with drinking reductions or abstinence
CBI Problem	PWH who maintained or increased UAU at visit 2 after receiving CBI Prime at visit 1	Discusses possible barriers to reduction, coping strategies, and the use of alcohol pharmacotherapy
CBI Prize	PWH who reduced UAU to lower risk use at visit 2	Reviews patients’ experiences in reducing their alcohol use, identifies successful strategies, reviews risky drinking, coping strategies, and provides an opportunity to set new drinking goals
CBT4CBT	PWH with moderate to severe AUD at visit 1 or visit 2	Online CBT program consisting of seven modules, 45 min each, that include refusal skills, recognizing and coping with cravings, addressing thoughts about alcohol use, patterns of thinking, and decision making around alcohol use
Alcohol pharmacotherapy	PWH with moderate to severe AUD at visit 1 or visit 2	FDA approved therapies for alcohol use, including naltrexone and acamprosate

*Stepped care procedure*: For PWH with UAU without moderate to severe AUD based on screening, CBI Prime will be delivered by computer or person based on clinic site or patient preference. For those with moderate to severe AUD, PWH will receive CBI Problem (computer or person delivered) tailored to higher alcohol use severity that includes information on pharmacotherapy. In addition, they are provided with three options: CBT4CBT, alcohol pharmacotherapy, or both. At their next scheduled clinic visit (within ~ 4–6 months), PWH will undergo the PRO, and they will receive a second intervention based on this follow-up AUDIT-C score. PWH who continue to drink at unhealthy levels will be stepped up to a higher level of care and receive CBI Problem that reinforces alcohol pharmacotherapy and addresses medication adherence barriers. They will also be offered CBT4CBT and alcohol pharmacotherapy (see Fig. 2). Those with alcohol below unhealthy levels receive a brief intervention with affirming messages via CBI Prize.

As part of the design of the trial, clinics can choose to offer flexible options for the delivery of alcohol EBIs including CBI, face-to-face with a provider, or telemedicine. These modalities will vary both within and between clinics dependent on clinic and patient preferences and capacity. A staff member selected by each site, who could be a social worker, behavioral health specialist, or another appropriate professional depending on the clinic’s structure and staff availability, will facilitate interventions. Clinic operations will be pragmatic, and thus, it is possible staff will turn over during the study period. During the practice facilitation period, the practice facilitator will be available to support sites through any barriers that arise, including staff turnover.

### Formative and summative evaluation

#### Overview

We will use mixed methods to conduct both formative and summative evaluations. These evaluations will support identification and addressing of implementation barriers in real time and will also be used to assess both implementation (reach, adoption, implementation, and maintenance) and effectiveness outcomes. The following data sources will be used: (1) surveys of staff and clinic leaders; (2) qualitative data from formative and summative evaluation activities throughout the implementation period, including interviews and participant observation of all implementation meetings between the practice coach and LIT, (3) patient-reported data (PROs) on alcohol use, receipt of alcohol-related care, and ART adherence, and (4) quantitative EHR data that measures receipt of alcohol-related care and HIV-related clinical outcomes including HIV viral load. The alcohol biomarker phosphatidyl ethanol (PEth) is collected by dried blood spot. Study outcomes are summarized in Table 3 below.

#### Data sources, collection, and measures

##### **Surveys of staff and clinic leaders**

During the pre-implementation phase, we will conduct surveys with clinic staff at each site to gather baseline information about their current alcohol care practices and tailor the practice facilitation intervention to their local context. Surveys will be administered electronically via REDCap during the pre-implementation phase [50, 51]. These surveys will comprise both structured and open-ended questions, aimed at understanding the staff’s knowledge, attitudes, beliefs, and practices.

##### **Qualitative data from formative and summative interviews and observations**

We will conduct semi-structured qualitative interviews during both the pre-implementation (formative) and sustainment (summative) phases with staff members from participating clinics. During the pre-implementation phase, we will ask about the staff’s experiences with alcohol EBIs, clinic processes, and clinical cultures. In the sustainment phase, semi-structured interviews will focus on clinic staff’s experiences with the clinical and implementation intervention and perspectives on needed supports to sustain stepped care and preferences for person or computer-delivered intervention. At both stages, we will employ a combination of purposive and snowball sampling informed by the LIT to identify potential interview participants, including individuals in clinical, administrative/support, and leadership roles. LIT teams will send recruitment emails to all eligible providers at their clinics and introduce the study/interview recruitment at onsite meetings, attended by clinic staff, to elicit interest equally across all providers. Snowball sampling will be used to identify potential participants that may have important perspectives and need additional recruitment outreach. The study team will also regularly review interview recruitment progress to assess for missing perspectives and will work with LIT teams to adjust recruitment strategies as needed. We anticipate reaching thematic saturation with a sample size of 21–30 participants (7–10 from each clinic). Potential interviewees will be identified and scheduled for phone interviews conducted by trained study staff. At the start of each interview, participants will be asked to provide verbal consent. The interviews, lasting approximately 30–45 min, will consist of open-ended questions and semi-structured follow-up questions guided by the domains of the CFIR. For sustainment period interviews, we will follow the same interview recruitment and data collection procedures described above with the goal of recruiting 5–7 clinical staff and 5–7 patients per site.

For patient interviews, LIT research staff will approach eligible patients after in-clinic visits to elicit interest in interview participation. Content will include experiences with intervention participation and explore preferences person or computer-delivery. Patients who agree to participate in a 60-minute study interview will then be contact via email to schedule the interview. All interviews will take place virtually, via the online platform Zoom, and participants will receive $75 as compensation for participating in this component of the study.

During the entire study period (pre-implementation, implementation, sustainment), we will conduct ***participant observation*** of all implementation meetings between the Practice Coach and LITs and clinic representatives. Similar to our prior studies [30, 52, 53], a trained qualitative researcher will take verbatim ethnographic fieldnotes of discussions that happen during implementation meetings. We will then use structured templates to summarize observed meeting data by site. Iterative summaries will be generated to identify barriers and facilitators by site, address barriers (e.g., staff turnover) and capitalize on facilitators (e.g. change in practice facilitator skills) in real time, and to assess implementation outcomes [54].

##### **Patient-reported data through the CNICS PRO**

Data on self-reported alcohol use (AUDIT-C, AUDIT, MINI) [38, 40, 55, 56], receipt of alcohol-related care (Treatment Service Review), and ART adherence (Self-rating and Visual Analog Scale) [57–61] will be extracted from the PROs. Specific to this implementation study, we have added an additional PRO question asking patients if they received an alcohol intervention since their last visit.

##### **Biomarkers**

Using dried blood spot collection, we will measure an alcohol biomarker, PEth. Because PEth is not part of routine care, participants will be compensated $10 for this procedure [43, 62].

##### **EHR data**

We will collect patient demographics, HIV1-RNA, CD4 cell count, and alcohol pharmacotherapy prescription from the EHR using the CNICS data collection infrastructure [36].

##### Study outcomes

Our study outcomes, guided by RE-AIM, are described in Table 3 below.

**Table 3:** Study outcomes using the RE-AIM framework

Dimension	Exemplar Use Defined by NCI	Present Study Measure
**R**each	• Percent of individuals who participate, among all patients	Percent of patients with UAU who receive an alcohol EBI
	• Characteristics of participants compared to non-participants or to target population	Above measure compared across racial/ethnic groups, gender, and clinical characteristics
**E**ffectiveness	• Measure of primary or broader outcomes	1) Reduced UAU (AUDIT-C score < 3/4 women/men)* 2) Viral suppression (HIV-RNA < 200) 3) ART adherence (> 90%) 4) PEth
**A**doption	• Use of qualitative methods to understand adoption at setting level • Use of qualitative methods to understand staff participation	Barriers and facilitators to delivering alcohol EBIs, intervention components important to enhancing delivery and site preferences related to selection of computer or person delivered intervention.
	• Percent of staff invited that participate	1) Provider report of delivering alcohol EBIs 2) Percent of providers prescribing AUD pharmacotherapy among all clinic providers
**I**mplementation	• Percent of intervention delivery or calls completed, etc. (e.g., adherence or consistency)	1) Percent of patients reporting receiving an alcohol intervention since their last visit among all responding to the PROs 2) Percent of alcohol treatment sessions completed
**M**aintenance	• Measure of primary outcome (with or without comparison to a public health goal) at ≥6mo follow-up after final intervention contact	Percent of patients reporting receiving an alcohol intervention in the past six months among all responding to PROs at six months post final contact with Practice Coach

#### Data analysis and interpretation

##### **Formative surveys**

 Survey responses gathered from clinical staff during the formative evaluation will be summarized overall and at the clinic level; cross tabs of key variables will be performed and summarized.

##### **Formative participant observations**

Participant observation notes will be analyzed using the Rapid Assessment Process described above to assess Adoption. Qualitative data will be coded using techniques from Grounded Theory and Template Analysis using CFIR domains as the analysis template and guide, as we have done in similar studies [41], and used to identify barriers and facilitators aligned with CFIR domains, address barriers (e.g., staff turnover) and capitalize on facilitators in real time, and to determine the effects of practice facilitation at sites.

##### **Formative and summative qualitative interviews**

All qualitative ***interviews*** will be de-identified, digitally recorded, and transcribed by an external source. The transcriptions will be reviewed for accuracy and analyzed using the Rapid Assessment Process [63–66]. This process will be conducted iteratively to identify emergent themes with the multidisciplinary research team, triangulate data with existing theoretical knowledge and our previous research regarding implementation of brief interventions, make mid-course corrections in data collection, and check for saturation of themes. Interviews from the summative phase will be analyzed using a combination of Rapid Assessment Process and thematic analysis and triangulated with other data sources (e.g., participant observations, survey, clinical data) to inform evaluation of Aim 2a and Aim 3.

## Reach and effectiveness evaluation (clinical measures of UAU and HIV care, Aim 2b)

Reach and Effectiveness will be assessed using an interrupted time series with synthetic control to evaluate whether practice facilitation is associated with our primary implementation outcome (percent of patients reporting receiving an alcohol intervention since their last visit among all responding to PROS across all CNICS sites) and with our secondary effectiveness outcomes (alcohol use, ART adherence, and viral suppression) [67, 68].

An interrupted time series design allows for conducting a rigorous naturalistic study, results can be displayed graphically to intuitively represent findings, historical trend is accounted for in the analytic model, and the size of the effect can be estimated at different times post intervention dissemination [69–71]. Addition of synthetic control can improve estimation of the counterfactual condition in which the intervention was not added to the specific HIV clinics by reducing selection bias and satisfying the parallel trends assumption, which is needed for controlled interrupted time series analyses [69].

The sample will include all patients with UAU seen at each of the CNICS sites during the study. The CNICS sites that did not have the intervention will be the control sites for the interrupted time series with synthetic control. The outcomes will be expressed as a proportion per month. Therefore, the denominator for all outcomes is all patients with UAU with a visit to the participating CNICS clinics for each month of the study period. Numerators for the effectiveness outcomes are defined as follows: (1) Implementation based on patient report (primary outcome); (2) AUDIT-C score ≤ 3 women and ≤ 4 men; (3) viral suppression as indicated by HIV RNA ≤ 200 copies; and (4) high adherence to ART defined as 90% on the visual analog scale.

The analysis will be stratified or adjusted by sex, race, and social determinants of health, including housing and income. Should one of the non-intervention CNICS sites be ineligible as a control (for instance, if it is implementing a different alcohol treatment initiative) and optimal weights for synthetic control cannot be determined, we will transition to a comparative interrupted time series. The sequential roll-out of the intervention offers added resilience against temporal secular trend threats. However, we will also model each site as a main effect prior to the intervention, as well as the main effect of time prior to the intervention, and the interaction between time and the intervention. This approach allows each site to have its own pre- and post-intervention slopes relative to its comparative synthetic control, thereby accounting for potential differences between the first, second, and third sites that could arise from secular time trends. To account for potential improvement in the practice coach’s skill over the sequential roll-out, we will also conduct the interrupted time series compared to synthetic control by site to assess for effect heterogeneity. This will be an exploratory analysis as this was not a part of our initial protocol.

## Power considerations

There are no formal power calculations for a comparative interrupted time series with a synthetic control. Inferential strength in synthetic control depends mainly on the quality of the pre-intervention match and not the number of units [72]. Furthermore, comparative interrupted time series uses the number of pre- and post-intervention time points as its effective sample size [73]. Our long pre-intervention series supports stable estimation of baseline trends and construction of a reliable synthetic counterfactual. Finally, the synthetic control’s ability to weight control sites to match intervention sites allows us to draw insights from previous power examinations for comparative interrupted time series. We have examined the autocorrelation between months across the intervention sites over the past two years. The autocorrelation was centered on 0, with slight variations by site and sex (ranging from − 0.13 to 0.15 with a standard error of approximately 0.24). Given this autocorrelation, we anticipate being able to detect an effect in the trend change between 0.25 and 0.5 standard deviations.

## Discussion

Despite the availability of EBIs for UAU that improve HIV treatment outcomes, the evidence to practice gap remains wide. To address this gap, this research uses a hybrid-3 implementation-effectiveness design to test whether practice facilitation can increase reach, effectiveness, adoption, implementation, and maintenance of stepped care delivery of alcohol EBIs in HIV clinics.

There are numerous strengths of the research proposed. Our study applies established implementation science frameworks, practices, and analytic approaches to improve knowledge on maximizing integration of established alcohol EBIs into HIV clinic settings. Our research is conducted in real-world HIV clinics and built into usual practice conditions, where validated screening for UAU occurs in the context of clinical visits. In addition, our study uses mixed-methods to understand barriers and facilitators to sustainable integration of alcohol EBIs. Our summative evaluation relies on novel contemporary advanced implementation science analytic approaches that combine familiar interrupted time series methods with novel synthetic controls, enabling us to assess the effect of the implementation intervention independent of other aspects that influence care.

This study will not be without limitations. CNICS clinics have built in data capture systems along with routine assessment of patient reported outcomes that facilitate screening and intervention, along with dedicated staff who can assist with positive screens. As such, these clinics are not likely representative of all HIV clinics, and the focus of practice facilitation activities may differ in non-CNICS settings. Further, while the quasi-experimental approach applies cutting edge implementation science methods to evaluate the intervention, it does not enable an unequivocal assessment of causality. However, a cluster randomized trial of the required size (32–40 clinics) would be extremely costly and likely infeasible, and the interrupted time series design with synthetic controls allows us to test whether the intervention is associated with improvements in both implementation and effectiveness outcomes. Additionally, in the event that a CNICS site cannot serve as a control (e.g., implementing a different alcohol treatment initiative) and optimal weights cannot be determined for synthetic control, we will move to a comparative interrupted time series. Finally, the sequential roll-out of the intervention strengthens the design by providing additional protection against threats to validity resulting from secular trends over time.

UAU among PWH disrupts optimal engagement in the HIV Care Continuum and, through it, affects viral suppression and transmission risk behaviors. With the availability of effective behavioral and pharmacological interventions for UAU, integration of alcohol EBIs into HIV clinical settings are a recommended component of comprehensive care [74]. As such, rigorously evaluating implementation strategies that may improve evidence-based alcohol screening and interventions in HIV clinical settings is essential to achieve US goals for the care of PWH and for ending the HIV epidemic.

## Data Availability

No datasets were generated or analysed during the current study.

## Declarations

### Regulatory approval

This study was approved by the Johns Hopkins University School of Medicine Institutional Review Board (IRB00303116) which serves as the single IRB for this multi-institutional collaboration. Collaborating institutions include the University of California, San Diego, University of Washington, Seattle, University of North Carolina, Chapel Hill and Fenway Community Health Center, Boston. All collaborating institutions have acknowledged and approved the study protocol through their respective reliance agreements with the Johns Hopkins University School of Medicine IRB. The IRB granted waivers of informed consent and HIPAA authorization to access secondary electronic health record data used to identify patients eligible for recruitment. Written informed consent is obtained from patients enrolled in the clinical trial, and verbal or written informed consent is obtained from interview participants, per approval from the IRB. All procedures adhere to the ethical standards outlined in the Declaration of Helsinki and relevant local regulatory requirements. The trial is registered with Clinicaltrials.gov, identifier: NCT05241990, date of submission 2/16/2022.

### Recruitment and timeline

Practice facilitation will begin at each of the three clinics in spring 2022, 2023, and 2024, respectively, followed by 3–6 months of preparation before enrollment commences. The preparation phase may differ between the clinics due to variation in preparedness and support needed to launch enrollment. Clinics will be given one year to enroll participants in the ARCH program, followed by 3–6 months of sustainment where LIT staff will continue to approach enrolled patients for follow-up visits. Recruitment for this study began on February 15, 2023. Key enrollment milestones include March–December 2023: Recruitment of participants from Site 1, target 100–150 participants; January–June 2024: Recruitment of participants from Site 2,target 100–150 participants; July 2024–December 2025: Recruitment of participants from Site 3, target 100–150 participants. Recruitment for this study was completed 1/12/2026.

## References

[1] Saitz R. Unhealthy Alcohol Use. N Engl J Med. 2005;352(6):596–607. 15703424 10.1056/NEJMcp042262

[2] Crane HM, McCaul ME, Chander G, Hutton H, Nance RM, Delaney JAC, et al. Prevalence and Factors Associated with Hazardous Alcohol Use Among Persons Living with HIV Across the US in the Current Era of Antiretroviral Treatment. AIDS Behav. 2017;21(7):1914–25. 28285434 10.1007/s10461-017-1740-7 PMC5628735

[3] Barai N, Monroe A, Lesko C, Lau B, Hutton H, Yang C, et al. The Association Between Changes in Alcohol Use and Changes in Antiretroviral Therapy Adherence and Viral Suppression Among Women Living with HIV. AIDS Behav. 2017;21(7):1836–45. 27752873 10.1007/s10461-016-1580-x PMC5393959

[4] Chander G, Lau B, Moore RD. Hazardous Alcohol Use. J Acquir immune Defic syndromes: JAIDS. 2006;43(4):411–7. 10.1097/01.qai.0000243121.44659.a4 PMC2704473 17099312

[5] Hutton HE, Lesko CR, Li X, Thompson CB, Lau B, Napravnik S, et al. Alcohol Use Patterns and Subsequent Sexual Behaviors Among Women, Men who have Sex with Men and Men who have Sex with Women Engaged in Routine HIV Care in the United States. AIDS Behav. 2019;23(6):1634–46. 30443807 10.1007/s10461-018-2337-5 PMC6830881

[6] Lesko CR, Lau B, Chander G, Moore RD. Time Spent with HIV Viral Load > 1500 Copies/mL Among Persons Engaged in Continuity HIV Care in an Urban Clinic in the United States, 2010–2015. AIDS Behav. 2018;22(11):3443–50. 29541913 10.1007/s10461-018-2085-6 PMC6467647

[7] Williams EC, McGinnis KA, Edelman EJ, Matson TE, Gordon AJ, Marshall BDL, et al. Level of Alcohol Use Associated with HIV Care Continuum Targets in a National U.S. Sample of Persons Living with HIV Receiving Healthcare. AIDS Behav. 2019;23(1):140–51. 29995206 10.1007/s10461-018-2210-6 PMC6344313

[8] Shield KD, Parry C, Rehm J. Chronic diseases and conditions related to alcohol use. Alcohol Res. 2013;35(2):155–73. 24881324 10.35946/arcr.v35.2.06 PMC3908707

[9] O’Connor EA, Perdue LA, Senger CA, Rushkin M, Patnode CD, Bean SI, et al. Screening and Behavioral Counseling Interventions to Reduce Unhealthy Alcohol Use in Adolescents and Adults: Updated Evidence Report and Systematic Review for the US Preventive Services Task Force. JAMA. 2018;320(18):1910–28.30422198 10.1001/jama.2018.12086

[10] Green PP, Cummings NA, Ward BW, McKnight-Eily LR. Alcohol screening and brief intervention: office-based primary care physicians, U.S., 2015–2016. Am J Prev Med. 2022;62(2):219 – 26. 10.1016/j.amepre.2021.07.013 PMC9080450 34774391

[11] Cook WK, Ye Y, Zhu Y, Karriker-Jaffe KJ, Mulia N. Trends and disparities in alcohol screening and brief counseling following the U.S. Affordable Care Act. Drug Alcohol Depend. 2025;268:112558. 39837225 10.1016/j.drugalcdep.2025.112558 PMC11879066

[12] Substance A, Mental Health Services A. Key substance use and mental health indicators in the United States: results from the 2017 national survey on drug use and health 2018 [updated October 22, 2020. Available from: https://store.samhsa.gov/product/Key-Substance-Use-and-Mental-Health-Indicators-in-the-United-States-Results-from-the-2017-National-Survey-on-Drug-Use-and-Health/SMA18-5068.

[13] Substance A, Mental Health Services A. Key Substance Use and Mental Health Indicators in the United States:Results from the 2024 National Survey on Drug Use and Health. (HHS Publication No. PEP25-07-007, NSDUH Series H-60). Center for Behavioral Health Statistics and Quality, Substance Abuse and Mental Health Services Administration. https://www.samhsa.gov/data/data-we%10collect/nsduh%10national%10survey%10drug%10use%10and%10health/national%10releases.

[14] Papas RK, Gakinya BN, Mwaniki MM, Lee H, Keter AK, Martino S, et al. A randomized clinical trial of a group cognitive–behavioral therapy to reduce alcohol use among human immunodeficiency virus-infected outpatients in western Kenya. Addiction. 2021;116(2):305–18. 32422685 10.1111/add.15112 PMC7671944

[15] Go VF, Hutton HE, Ha TV, Chander G, Latkin CA, Mai NVT, et al. Effect of 2 Integrated Interventions on Alcohol Abstinence and Viral Suppression Among Vietnamese Adults With Hazardous Alcohol Use and HIV. JAMA Netw Open. 2020;3(9):e2017115. 32945875 10.1001/jamanetworkopen.2020.17115 PMC7501538

[16] Chander G, Hutton HE, Lau B, Xu X, McCaul ME. Brief Intervention Decreases Drinking Frequency in HIV-Infected, Heavy Drinking Women. JAIDS J Acquir Immune Defic Syndr. 2015;70(2):137–45. 25967270 10.1097/QAI.0000000000000679 PMC4634011

[17] Edelman EJ, Maisto SA, Hansen NB, Cutter CJ, Dziura J, Deng Y, et al. Integrated stepped alcohol treatment for patients with HIV and alcohol use disorder: a randomised controlled trial. lancet. 2019;6(8):e509–17. 10.1016/S2352-3018(19)30076-1 PMC7161741 31109915

[18] Satre DD, Leibowitz AS, Leyden W, Catz SL, Hare CB, Jang H, et al. Interventions to Reduce Unhealthy Alcohol Use among Primary Care Patients with HIV: the Health and Motivation Randomized Clinical Trial. J Gen Intern Med. 2019;34(10):2054–61. 31187344 10.1007/s11606-019-05065-9 PMC6816606

[19] Kahler CW, Pantalone DW, Mastroleo NR, Liu T, Bove G, Ramratnam B, et al. Motivational interviewing with personalized feedback to reduce alcohol use in HIV-infected men who have sex with men: A randomized controlled trial. J Consult Clin Psychol. 2018;86(8):645–56. 30035581 10.1037/ccp0000322 PMC6061969

[20] Springer SA, Di Paola A, Azar MM, Barbour R, Biondi BE, Desabrais M, et al. Extended-Release Naltrexone Improves Viral Suppression Among Incarcerated Persons Living With HIV With Opioid Use Disorders Transitioning to the Community: Results of a Double-Blind, Placebo-Controlled Randomized Trial. JAIDS J Acquir Immune Defic Syndr. 2018;78(1):43–53. 29373393 10.1097/QAI.0000000000001634 PMC5889326

[21] Hasin DS, Aharonovich E, O’Leary A, Greenstein E, Pavlicova M, Arunajadai S, et al. Reducing heavy drinking in < scp>HIV primary care: a randomized trial of brief intervention, with and without technological enhancement. Addiction. 2013;108(7):1230–40. 23432593 10.1111/add.12127 PMC3755729

[22] McCaul ME, Hutton HE, Cropsey KL, Crane HM, Lesko CR, Chander G, et al. Decreased alcohol consumption in an implementation study of computerized brief intervention among HIV patients in clinical care. AIDS Behav. 2021;25(12):4074–84. 33993353 10.1007/s10461-021-03295-9 PMC8594281

[23] Edelman EJ, Dziura J, Esserman D, Porter E, Becker WC, Chan PA, et al. Working with HIV clinics to adopt addiction treatment using implementation facilitation (WHAT-IF?): Rationale and design for a hybrid type 3 effectiveness-implementation study. Contemp Clin Trials. 2020;98:106156. 32976995 10.1016/j.cct.2020.106156 PMC7511156

[24] Chander G, Monroe AK, Crane HM, Hutton HE, Saag MS, Cropsey K, et al. HIV primary care providers–Screening, knowledge, attitudes and behaviors related to alcohol interventions. Drug Alcohol Depend. 2016;161:59–66. 26857898 10.1016/j.drugalcdep.2016.01.015 PMC4841449

[25] Baskerville NB, Liddy C, Hogg W. Systematic Review and Meta-Analysis of Practice Facilitation Within Primary Care Settings. Annals Family Med. 2012;10(1):63–74. 10.1370/afm.1312 PMC3262473 22230833

[26] Agency for Healthcare R. Quality. Chapter 2: What is Practice Coaching? [updated October 28, 2020. Available from: https://www.ahrq.gov/ncepcr/care/chronic-manual/about.html.

[27] The Commonwealth F. Facilitating improvement in primary care: the promise of practice coaching 2012 [updated November 3, 2020. Available from: https://www.commonwealthfund.org/publications/issue-briefs/2012/jun/facilitating-improvement-primary-care-promise-practice-coaching. 22712103

[28] University of Washington AC. CHAMP Clinical Trial [updated October 28. 2020. Available from: http://aims.uw.edu/champ-clinical-trial.

[29] Frost MC, Ioannou GN, Tsui JI, Edelman EJ, Weiner BJ, Fletcher OV, et al. Practice facilitation to implement alcohol-related care in Veterans Health Administration liver clinics: a study protocol. Implement Sci Commun. 2020;1(1). 10.1186/s43058-020-00062-0 PMC7393339 32835226

[30] Glass JE, Bobb JF, Lee AK, Richards JE, Lapham GT, Ludman E, et al. Study protocol: a cluster-randomized trial implementing sustained patient-centered alcohol-related care (SPARC trial). Implement Sci. 2018;13(1). 10.1186/s13012-018-0795-9 PMC6080376 30081930

[31] Lee AK, Bobb JF, Richards JE, Achtmeyer CE, Ludman E, Oliver M, et al. Integrating Alcohol-Related Prevention and Treatment Into Primary Care: A Cluster Randomized Implementation Trial. JAMA Intern Med. 2023;183(4):319–28. 36848119 10.1001/jamainternmed.2022.7083 PMC9972247

[32] Huffstetler AN, Villalobos G, Webel B, Rockwell MS, Funk A, Sabo RT, et al. Practice Facilitation to Address Unhealthy Alcohol Use in Primary Care: A Cluster Randomized Clinical Trial. JAMA Health Forum. 2024;5(8):e242371. 39120895 10.1001/jamahealthforum.2024.2371 PMC11316228

[33] Glasgow RE, Vogt TM, Boles SM. Evaluating the public health impact of health promotion interventions: the RE-AIM framework. Am J Public Health. 1999;89(9):1322–7. 10474547 10.2105/ajph.89.9.1322 PMC1508772

[34] Damschroder LJ, Aron DC, Keith RE, Kirsh SR, Alexander JA, Lowery JC. Fostering implementation of health services research findings into practice: a consolidated framework for advancing implementation science. Implement Sci. 2009;4(1):50. 19664226 10.1186/1748-5908-4-50 PMC2736161

[35] Pinnock H, Barwick M, Carpenter CR, Eldridge S, Grandes G, Griffiths CJ, et al. Standards for reporting implementation studies (StaRI) Statement. BMJ. 2017:i6795. 10.1136/bmj.i6795 PMC5421438 28264797

[36] Kitahata MM, Rodriguez B, Haubrich R, Boswell S, Mathews WC, Lederman MM, et al. Cohort profile: the Centers for AIDS Research Network of Integrated Clinical Systems. Int J Epidemiol. 2008;37(5):948–55. 18263650 10.1093/ije/dym231 PMC2597168

[37] Crane HM, Lober W, Webster EW, Harrington RD, Crane PK, Davis TE, et al. Routine collection of patient-reported outcomes in an HIV clinic setting: the first 100 patients - PubMed. Curr HIV Res. 2007;5(1). 10.2174/157016207779316369 17266562

[38] Sheehan DV, Lecrubier Y, Sheehan KH, Amorim P, Janavs J, Weiller E, et al. The Mini-International Neuropsychiatric Interview (M.I.N.I.): the development and validation of a structured diagnostic psychiatric interview for DSM-IV and ICD-10. J Clin Psychiatry. 1998;59(Suppl 20):22–33. quiz 4–57. 9881538

[39] Bradley KA, Bush KR, Epler AJ, Dobie DJ, Davis TM, Sporleder JL, et al. Two Brief Alcohol-Screening Tests From the Alcohol Use Disorders Identification Test (AUDIT). Arch Intern Med. 2003;163(7):821. 12695273 10.1001/archinte.163.7.821

[40] Bush K. The AUDIT Alcohol Consumption Questions (AUDIT-C)<subtitle > An Effective Brief Screening Test for Problem Drinking</subtitle >. Arch Intern Med. 1998;158(16):1789. 9738608 10.1001/archinte.158.16.1789

[41] Williams EC, Young JP, Achtmeyer CE, Hendershot CS. Primary Care Providers’ Interest in Using a Genetic Test to Guide Alcohol Use Disorder Treatment. J Subst Abuse Treat. 2016;70:14–20. 27692183 10.1016/j.jsat.2016.07.009

[42] National Cancer I. Measuring the use of the RE-AIM model dimension items checklist [updated November 3, 2020. Available from: http://cancercontrol.cancer.gov/IS/.

[43] Bajunirwe F, Haberer JE, Boum Y, Hunt P, Mocello R, Martin JN, et al. Comparison of Self-Reported Alcohol Consumption to Phosphatidylethanol Measurement among HIV-Infected Patients Initiating Antiretroviral Treatment in Southwestern Uganda. PLoS ONE. 2014;9(12):e113152. 25436894 10.1371/journal.pone.0113152 PMC4249861

[44] Smith JD, Li DH, Rafferty MR. The implementation research logic model: a method for planning, executing, reporting, and synthesizing implementation projects. Implement Sci. 2020;15(1). 10.1186/s13012-020-01041-8 PMC7523057 32988389

[45] Landes SJ, McBain SA, Curran GM. An introduction to effectiveness-implementation hybrid designs. Psychiatry Res. 2019;280:112513. 31434011 10.1016/j.psychres.2019.112513 PMC6779135

[46] Kilbourne AM, Geng E, Eshun-Wilson I, Sweeney S, Shelley D, Cohen DJ, et al. How does facilitation in healthcare work? Using mechanism mapping to illuminate the black box of a meta-implementation strategy. Implement Sci Commun. 2023;4(1). 10.1186/s43058-023-00435-1 PMC10190070 37194084

[47] Ritchie MJDK, Miller CJ, Smith JL, Oliver KA, Kim B, Connolly SL, Woodward E, Ochoa-Olmos T, Day S, Lindsay JA, Kirchner JE. Implementation strategies & tools – VA QUERI. QUERI - US Department of Veterans Affairs. 2024.

[48] Albers B, Metz A, Burke K, Bührmann L, Bartley L, Driessen P, et al. Implementation Support Skills: Findings From a Systematic Integrative Review. Res social work Pract. 2021;31(2):147–70. 10.1371/journal.pone.0267533 PMC9094539 35544529

[49] Kiluk BD, Benitez B, Devito EE, Frankforter TL, Lapaglia DM, O’Malley SS, et al. A Digital Cognitive Behavioral Therapy Program for Adults With Alcohol Use Disorder. JAMA Netw Open. 2024;7(9):e2435205. 39325452 10.1001/jamanetworkopen.2024.35205 PMC11428014

[50] Harris PA, Taylor R, Minor BL, Elliott V, Fernandez M, O’Neal L, et al. The REDCap consortium: Building an international community of software platform partners. J Biomed Inform. 2019;95:103208. 31078660 10.1016/j.jbi.2019.103208 PMC7254481

[51] Harris PA, Taylor R, Thielke R, Payne J, Gonzalez N, Conde JG. Research electronic data capture (REDCap)—A metadata-driven methodology and workflow process for providing translational research informatics support. J Biomed Inform. 2009;42(2):377–81. 18929686 10.1016/j.jbi.2008.08.010 PMC2700030

[52] Austin EJ, Chen J, Briggs ES, Ferro L, Barry P, Heald A et al. Integrating opioid use disorder treatment into primary care settings - PubMed. JAMA Netw open. 08/01/2023;6(8). 10.1001/jamanetworkopen.2023.28627 PMC10422185 37566414

[53] Austin EJ, Briggs ES, Ferro L, Barry P, Heald A, Curran GM et al. Integrating routine screening for opioid use disorder into primary care settings: experiences from a national cohort of clinics - PubMed. J Gen Intern Med. 2023;38(2). 10.1007/s11606-022-07675-2 PMC9132563 35614169

[54] Williams EC, Frost MC, Danner AN, Lott AMK, Achtmeyer CE, Hood CL, et al. The Only Reason I Am Willing to Do It at All: Evaluation of VA’s SUpporting Primary care Providers in Opioid Risk reduction and Treatment (SUPPORT) Center. J Addict Med. 2024;18(3):248–55. 38385548 10.1097/ADM.0000000000001277

[55] Saunders JB, Aasland OG, Babor TF, de la Fuente JR, Grant M. Development of the Alcohol Use Disorders Identification Test (AUDIT): WHO Collaborative Project on Early Detection of Persons with Harmful Alcohol Consumption–II. Addiction. 1993;88(6):791–804. 8329970 10.1111/j.1360-0443.1993.tb02093.x

[56] McLellan AT, Alterman AI, Cacciola J, Metzger D, OʼBrien CP. A New Measure of Substance Abuse Treatment Initial Studies of the Treatment Services Review. J Nerv mental disease. 1992;180(2):101–10. 10.1097/00005053-199202000-00007 1737971

[57] Feldman BJ, Fredericksen RJ, Crane PK, Safren SA, Mugavero MJ, Willig JH, et al. Evaluation of the Single-Item Self-Rating Adherence Scale for Use in Routine Clinical Care of People Living with HIV. AIDS Behav. 2013;17(1):307–18. 23108721 10.1007/s10461-012-0326-7 PMC3549002

[58] Crane HM, Nance RM, Delaney JAC, Fredericksen RJ, Church A, Simoni JM, et al. A Comparison of Adherence Timeframes Using Missed Dose Items and Their Associations with Viral Load in Routine Clinical Care: Is Longer Better? AIDS Behav. 2017;21(2):470–80. 27714525 10.1007/s10461-016-1566-8 PMC5290185

[59] Lu M, Safren SA, Skolnik PR, Rogers WH, Coady W, Hardy H, et al. Optimal Recall Period and Response Task for Self-Reported HIV Medication Adherence. AIDS Behav. 2008;12(1):86–94. 17577653 10.1007/s10461-007-9261-4

[60] Simoni JM, Kurth AE, Pearson CR, Pantalone DW, Merrill JO, Frick PA. Self-Report Measures of Antiretroviral Therapy Adherence: A Review with Recommendations for HIV Research and Clinical Management. AIDS Behav. 2006;10(3):227–45. 16783535 10.1007/s10461-006-9078-6 PMC4083461

[61] Amico KR, Fisher WA, Cornman DH, Shuper PA, Redding CG, Konkle-Parker DJ, et al. Visual Analog Scale of ART Adherence. J Acquir immune Defic syndromes: JAIDS. 2006;42(4):455–9. 10.1097/01.qai.0000225020.73760.c2 16810111

[62] Couture M-C, Page K, Sansothy N, Stein E, Vun MC, Hahn JA. High prevalence of unhealthy alcohol use and comparison of self-reported alcohol consumption to phosphatidylethanol among women engaged in sex work and their male clients in Cambodia. Drug Alcohol Depend. 2016;165:29–37. 27251102 10.1016/j.drugalcdep.2016.05.011 PMC5565395

[63] Creswell JW. Qualitative inquiry and research design. choosing among five approaches; 2007.

[64] Elo S, Kyngäs H. The qualitative content analysis process. J Adv Nurs. 2008;62(1):107–15. 18352969 10.1111/j.1365-2648.2007.04569.x

[65] Rowman L. Rapid assessment process: an introduction 2008 [updated October 28, 2020. Available from: https://rowman.com/ISBN/9780759117020/Rapid-Assessment-Process-An-Introduction.

[66] A H. Tips for Speeding Up Qualitative Projects2014.

[67] Degli Esposti M, Spreckelsen T, Gasparrini A, Wiebe DJ, Bonander C, Yakubovich AR, et al. Can synthetic controls improve causal inference in interrupted time series evaluations of public health interventions? Int J Epidemiol. 2020;49(6):2010–20. 10.1093/ije/dyaa152 33005920

[68] Linden A. Combining synthetic controls and interrupted time series analysis to improve causal inference in program evaluation. J Eval Clin Pract. 2018;24(2):447–53. 29356225 10.1111/jep.12882

[69] Kontopantelis E, Doran T, Springate DA, Buchan I, Reeves D. Regression based quasi-experimental approach when randomisation is not an option: interrupted time series analysis. BMJ. 2015;350(jun09 5):h2750–h. 26058820 10.1136/bmj.h2750 PMC4460815

[70] Bernal JL, Cummins S, Gasparrini A. Interrupted time series regression for the evaluation of public health interventions: a tutorial. Int J Epidemiol. 2017;46(1):348–55. 27283160 10.1093/ije/dyw098 PMC5407170

[71] Wagner AK, Soumerai SB, Zhang F, Ross-Degnan D. Segmented regression analysis of interrupted time series studies in medication use research. J Clin Pharm Ther. 2002;27(4):299–309. 12174032 10.1046/j.1365-2710.2002.00430.x

[72] Bouttell J, Craig P, Lewsey J, Robinson M, Popham F. Synthetic control methodology as a tool for evaluating population-level health interventions. J Epidemiol Community Health. 2018;72(8):673–8. 29653993 10.1136/jech-2017-210106 PMC6204967

[73] Zhang F, Wagner AK, Ross-Degnan D. Simulation-based power calculation for designing interrupted time series analyses of health policy interventions. J Clin Epidemiol. 2011;(11):1252–61. 10.1016/j.jclinepi.2011.02.007 21640554

[74] Health USDo, Human S. Guidelines for the use of antiretroviral agents in adults and adolescents with HIV. U.S. Department of Health and Human Services; 2024.

